# Kinetic, Thermodynamic, and Structural Analysis of Drug Resistance Mutations in Neuraminidase from the 2009 Pandemic Influenza Virus

**DOI:** 10.3390/v10070339

**Published:** 2018-06-21

**Authors:** Jana Pokorná, Petr Pachl, Elena Karlukova, Jakub Hejdánek, Pavlína Řezáčová, Aleš Machara, Jason Hudlický, Jan Konvalinka, Milan Kožíšek

**Affiliations:** 1 The Czech Academy of Sciences, Institute of Organic Chemistry and Biochemistry, Flemingovo n. 2, 166 10 Prague 6, Czech Republic; jana.pokorna@uochb.cas.cz (J.P.); petr.pachl@uochb.cas.cz (P.P.); karlukova@email.cz (E.K.); jakub.hejdanek@uochb.cas.cz (J.H.); pavlina.rezacova@uochb.cas.cz (P.Ř.); ales.machara@uochb.cas.cz (A.M.); j.r.hudlicky@gmail.com (J.H.); 2 Faculty of Food and Biochemical Technology, University of Chemistry and Technology, Technická 5, 166 28 Prague 6, Czech Republic; 3 Department of Biochemistry, Faculty of Science, Charles University, Hlavova 8, 128 00 Prague 2, Czech Republic; 4 The Czech Academy of Sciences, Institute of Molecular Genetics, Vídeňská 1083, 140 00 Prague 4, Czech Republic; 5 Department of Organic Chemistry, Faculty of Science, Charles University, Hlavova 8, 128 00 Prague 2, Czech Republic

**Keywords:** influenza neuraminidase, resistance, oseltamivir, isothermal titration calorimetry, crystal structure

## Abstract

Neuraminidase is the main target for current influenza drugs. Reduced susceptibility to oseltamivir, the most widely prescribed neuraminidase inhibitor, has been repeatedly reported. The resistance substitutions I223V and S247N, alone or in combination with the major oseltamivir-resistance mutation H275Y, have been observed in 2009 pandemic H1N1 viruses. We overexpressed and purified the ectodomain of wild-type neuraminidase from the A/California/07/2009 (H1N1) influenza virus, as well as variants containing H275Y, I223V, and S247N single mutations and H275Y/I223V and H275Y/S247N double mutations. We performed enzymological and thermodynamic analyses and structurally examined the resistance mechanism. Our results reveal that the I223V or S247N substitution alone confers only a moderate reduction in oseltamivir affinity. In contrast, the major oseltamivir resistance mutation H275Y causes a significant decrease in the enzyme’s ability to bind this drug. Combination of H275Y with an I223V or S247N mutation results in extreme impairment of oseltamivir’s inhibition potency. Our structural analyses revealed that the H275Y substitution has a major effect on the oseltamivir binding pose within the active site while the influence of other studied mutations is much less prominent. Our crystal structures also helped explain the augmenting effect on resistance of combining H275Y with both substitutions.

## 1. Introduction

Influenza is an acute respiratory viral infection that can cause serious complications and death, especially among elderly and the immunocompromised individuals. The most common circulating strains that infect humans are A (H1N1), A (H3N2), and B influenza viruses [1]. Annual seasonal flu epidemics, resulting on average in 3 to 5 million cases of severe disease and approximately 250,000 to 500,000 deaths worldwide [2], present a constant global threat to human health with a considerable socioeconomic burden. In addition, antigenically novel influenza A viruses emerge at irregular intervals. If the new viral strain transmits readily from person to person, it can quickly spread on a worldwide scale [3]. H1N1 viral subtypes have caused several influenza pandemics, including the most severe—the 1918 Spanish flu pandemic with more than 50 million victims—and the recent 2009 swine flu pandemic [4,5].

Vaccination remains the most effective strategy for influenza prevention. However, vaccine efficacy can be compromised by antigenic mismatch between the vaccine components and new, serologically distinct viral variants [6]. Other potential problems include suboptimal immunization in target patient groups and limited time for vaccine manufacturing at the beginning of a new outbreak of a pandemic strain [7]. In these cases, direct antiviral medication is an important part of defense, and it can be life-saving, especially for immunocompromised patients [8].

The administration of the first class anti-influenza drugs targeting the M2 channel is no longer recommended since most of circulating influenza A strains are resistant towards these inhibitors. Current influenza drugs target neuraminidase (NA), which plays an essential role in viral replication. NA is a homotetrameric transmembrane glycoprotein responsible for cleavage of sialic acid from the host cell membrane [9,10]. NA sequences that predominate in human influenza viruses fall into two distinct groups, called N1 and N2 [11]. N1 and N2 share 94% amino acid sequence identity in the active site, and 45% identity and 60% similarity overall [12]. The N1 NAs contain an additional cavity adjacent to the active site that has not been observed in N2 NA structures [13]. By blocking NA enzymatic activity, neuraminidase inhibitors (NAIs) prevent new viral particles from being released from the surface of an infected cell, thus stopping the spread of virus in the airways [14,15]. In addition, they decrease the virus’ ability to pass through mucus, which limits its ability to reach and infect epithelial cells [16].

At present, three NAIs are licensed worldwide for therapeutic and prophylactic use—inhaled zanamivir [17], marketed as Relenza^TM^; oral oseltamivir (Tamiflu^TM^) [18], the most widely prescribed NAI; and intravenous peramivir (Rapiacta^TM^) [19]. Another NAI called laninamivir (Inavir^TM^), is authorized for use in Japan, where it was originally developed [20]. Design of these NAIs is a classic example of structure-based drug discovery, guided by the first NA crystal structures [10,21,22]. The overall scaffolds of these inhibitors closely resemble the natural NA cleavage product, sialic acid. Neuraminidase binds and then cleaves sialic acid from glycan structures on the surface of the host cells and its inhibitors resemble this natural ligand. Taking into consideration the highly conserved active site sequence across all A and B NA subtypes, inhibitor-resistant mutants have been proposed to be enzymatically compromised and thus less viable [23].

However, drug-resistant viruses emerge readily because of the high mutation rate of viral RNA-dependent RNA polymerase [24] and frequent reassortment of genetic segments [25]. In the past, these variants were detected sporadically and did not spread [26]. However, as early as the 2007–2008 winter season, oseltamivir-resistant A(H1N1) viruses bearing a H275Y mutation in NA (N1 numbering) were reported in Europe at an unexpectedly high level (0% to 68%, by country) despite low antiviral drug use, and became widespread [27]. Surprisingly, resistance to oseltamivir was detected not only during treatment and prophylaxis, but also in influenza virus variants in untreated individuals. Evolution of the H275Y mutation in seasonal H1N1 was enabled by the secondary “permissive” mutations R222Q and V234M, which both individually and together increased viral fitness and transmissibility [28]. The mutation of His to Tyr at position 275 conferred a 750-fold reduction in susceptibility to oseltamivir and a 260-fold reduction in susceptibility to peramivir, based on a fluorometric inhibition assay (strain A/WSN/1933 (H1N1)) [29]. The structural basis of resistance conferred by the H275Y mutation has been described previously on the background of H5N1 avian influenza NA [30]. Briefly, the bulkier, polar tyrosine side chain repels the carboxyl group of the active site E277, which perturbs its reorientation needed for oseltamivir binding. The hydrophobic pentoxyl group of oseltamivir is in turn pushed out of the active site towards the I223 pocket, and the binding of oseltamivir is weakened. Computational studies have suggested an important role for the penetrating water molecules at the entrance to the active site of mutated NA; they may mediate a change in the electrostatic potential caused by a spatial reorganization of the amino acids surrounding the binding cavity [31,32]. In addition, a loss of the hydrogen bond between R152 and oseltamivir in the H275Y mutant may contribute to the decrease in susceptibility to this inhibitor [12].

More recently, I223V and S247N substitutions have been observed in 2009 pandemic H1N1 viruses [33,34,35,36]. The decrease in sensitivity to oseltamivir conferred by these single mutations alone is moderate, but when combined with the H275Y mutation, the double mutants have a greatly enhanced oseltamivir-resistant phenotype (1733-fold for H275Y/I223V and 7000-fold for H275Y/S247N compared to the recombinant wt virus). *In vitro* experiments by Pizzorno and coworkers suggested that addition of the I223V substitution to a H275Y recombinant mutant restored the slightly decreased replicative capacity caused by the H275Y mutation [33]. On the other hand, computer-simulated competition experiments showed a higher relative fitness for the H275Y single mutant over the I223V mutant in the presence of oseltamivir [37]. Position 223 of N1 NA is considered to be a ‘hotspot’ for substitutions, because I223 seems not to be critical for substrate binding, and its contacts with oseltamivir and zanamivir are more favorable than its substrate contacts [12,38,39]. Indeed, multiple mutations at this position can reduce the affinity of NA to oseltamivir without compromising viral fitness [38,40,41,42]. Similar features have been proposed for residue 247 [12]; however, fewer drug resistance mutations have been reported at this site [34,42]. Although residues in the active site of N1 and N2 subtypes of influenza type A share 94% identity, the patterns of resistance mutations for N1 and N2 NA are different (12). While the H275Y and S247N mutations are selected only in the N1 subtype, multiple substitutions at I223 are not type- or subtype-specific and confer oseltamivir resistance on N1, N2, and B NAs [38,43].

We performed kinetic, thermodynamic, and X-ray analyses to evaluate the influence of H275Y, I223V, and S247N mutations on the sensitivity of NA from 2009 pandemic influenza (NA2009) to oseltamivir. In agreement with previous work by others [33,34,35,36], we found that the I223V or S247N amino acid substitution alone confers only a mild decrease in oseltamivir affinity to NA2009, as suggested by our kinetic and isothermal titration calorimetry (ITC) analyses [44]. In contrast, the major NA2009 mutation, H275Y, causes a significant reduction in the enzyme’s ability to bind oseltamivir, as shown by detailed thermodynamic analyses and kinetic assays. Its combination with the I223V or S247N mutation leads to substantial impairment of oseltamivir’s inhibition potency. Our structural analyses explain this effect by a shift in position of E277, which interacts directly with oseltamivir, caused by the H275Y substitution. Compared to the H275Y single mutant, the double mutants underwent minor additional structural changes in protein-inhibitor contacts or waters that have an effect on increased resistance.

## 2. Materials and Methods 

### 2.1. Cloning, Expression, and Purification of Recombinant NA2009 Variants

DNA encoding the neuraminidase ectodomain (residues 82 to 469) from the A/California/07/2009 (H1N1) influenza virus was prepared by GenScript USA Inc. (Genbank Source Sequence CY121682, Piscataway, NJ, USA).

To obtain neuraminidases with amino acid substitutions, site-directed mutagenesis was performed. The sequences of the primers used for mutagenesis were as follows: H275Y—5′CCCTAATTATTACTATGAGGAAT3′ and 5′ATTCCTCATAGTAATAATTAGGG3′; I223V—5′GGAGAAACAATGTATTGAGAACAC3′ and 5′GTGTTCTCAATACATTGTTTCTCC3′; S247N—5′CGATGGACCAAATAATGGACAGG3′ and 5′CCTGTCCATTATTTGGTCCATCG3′.

Each resulting DNA sequence was inserted into the pMT/BiP/V5-HisA vector (Invitrogen, Carlsbad, CA, USA) with an N-terminal tag containing two Strep-tags, a FLAG tag, and a thrombin cleavage site. These constructs were used to transfect Drosophila Schneider S2 cells (Invitrogen), and large-scale expression was performed as previously described [45]. The recombinant neuraminidase expressed into cell culture medium was subsequently purified using one-step purification on Strep-Tactin agarose resin (IBA GmbH) [46,47]. First, Strep-Tactin resin was equilibrated in buffer W (0.1 M Tris-HCl, pH 8.0, 0.15 M NaCl), and medium with added biotin blocking solution (BioLock, IBA Lifesciences, Göttingen Germany) was applied. The matrix with bound tagged protein was thoroughly washed with buffer W, and elution was performed with buffer W containing 10 mM desthiobiotin. The resin was regenerated with buffer W containing 1 mM 2-(4-hydroxyphenylazo)benzoic acid (Sigma-Aldrich, St. Louis, MO, USA) and stored at 4 °C for later use.

The purification process was monitored by SDS PAGE and western blot using murine monoclonal anti-FLAG M2-peroxidase antibody clone M2 (Sigma-Aldrich). The N-terminal tag was removed by cleavage with thrombin protease immobilized on agarose beads (Sigma-Aldrich). Both expression and purification conditions were kept the same for all neuraminidase mutants.

### 2.2. Enzyme Kinetics of Recombinant NA2009 Variants

Enzyme kinetic parameters (*K*_m_ and *k*_cat_) and inhibition constants (*K*_i_) were determined by fluorometric assay using 2′-(4-methylumbelliferyl)-α-d-*N*-acetylneuraminic acid (4-MUNANA, Sigma-Aldrich) as a substrate [44]. Substrate cleavage was monitored using an Infinite M1000 reader (TECAN) with excitation/emission wavelengths set at 355/450 nm.

The 40 µL reactions contained 17 nM wt NA2009; 200 nM H275Y, S247N, H275Y/I223V, or H275Y/S247N NA2009; or 53 nM I223V NA2009 (calculated for monomer) and increasing substrate concentrations. The reactions were performed for 20 min at 37 °C and terminated by addition of 40 µL of 1 M sodium carbonate.

The inhibition constants for complexes of neuraminidases with oseltamivir carboxylate were determined by measuring the reduction in the substrate hydrolysis rate caused by different inhibitor concentrations. The data were analyzed using the equation for competitive inhibition according to Williams and Morrison [48] or Dixon analysis, when the *K*_i_ value was expected to be above 100 nM [49].

### 2.3. Isothermal Titration Calorimetry

The binding of oseltamivir carboxylate to the catalytic domain of neuraminidase was monitored using a VP-ITC microcalorimeter (MicroCal Inc., Malvern Instruments Ltd., Malvern, UK) at 25 °C in 50 mM MES buffer, pH 6.15, containing 150 mM NaCl and 10 mM CaCl_2_. The exact concentrations of proteins were determined by HPLC amino acid analysis. The inhibitor concentration was determined by elemental analysis. In a 1.43 mL sample cell, protein samples were titrated stepwise with 9 µL injections of inhibitor solution until saturation was achieved. The following protein and inhibitor concentrations were used: 6.2 µM I223V NA2009 or 8.7 µM S247N NA2009 with 125 µM oseltamivir, and 9.6 µM H275Y NA2009 with 180 µM oseltamivir. To discern whether inhibitor binding was accompanied by proton transfer, titrations were performed in two buffers with different ionization enthalpies (MES and ACES) [50]. All experiments were accompanied by controls in which ligand was injected into buffer alone to determine dilution heats. Thermodynamic parameters were determined in MicroCal software implemented in Origin 7.0 (MicroCal Inc., Malvern Instruments Ltd., Malvern, UK).

### 2.4. Protein Crystallography

The complexes for crystallization were prepared by mixing proteins in 5 mM Tris-HCl, pH 8.0, with oseltamivir carboxylate. A 3-fold molar excess of oseltamivir was used for the I223V, S247N, and S247N/H275Y mutants; a 12-fold excess for the H275Y mutant; and a 10-fold excess for the I223V/H275Y mutant. Protein-oseltamivir complexes were concentrated by ultrafiltration to a final concentration of 7–8 mg/mL. Crystals were grown using the hanging-drop vapor diffusion method at 19 °C. Drops consisted of 1 µL neuraminidase-oseltamivir carboxylate complex and 1 µL of reservoir solution. The reservoir solutions for the NA2009 mutants were as follows: I223V–0.1 M HEPES pH 6.75, 7% PEG 8000; S247N–0.1 M HEPES pH 6.7, 8.5% PEG 8000; H275Y–0.1 M HEPES pH 7.0, 8% PEG 8000; I223V/H275Y–0.1 M HEPES pH 7.0, 7% PEG 8000; and S247N/H275Y–0.1 M HEPES pH 7.5, 5% PEG 8000. All crystals were transferred into cryoprotectant consisting of reservoir solution supplemented with 20% (*v*/*v*) ethylene glycol and flash-cooled in liquid nitrogen.

### 2.5. Data Collection and Structure Determination

Complete datasets were collected at 100 K at beamline MX14.1 of BESSY, Berlin, Germany [51]. The dataset was processed using XDS, XDSGUI, and XDSAPP [52]. The crystal parameters and data collection statistics are listed in Table 1.

The structure was determined by molecular replacement with the program Molrep [55] using the crystal structure of wild-type NA2009 complexed with tamiphosphor as a search model [56]. Model refinement was performed using the program REFMAC 5.7.0032 [57] from the CCP4 package [58] in combination with manual adjustments in Coot software [59]. Oseltamivir was modelled after complete refinement of the protein chains and solvent model. The Molprobity server [60] was used to evaluate the final model quality. The final refinement statistics are summarized in Table 1. The structures were analyzed using the programs lsqkab (superpose) [61], baverage, and contact from the CCP4 package [58].

### 2.6. PDB Accession Codes

Atomic coordinates and structure factors have been deposited in the PDB database under accession codes 5NWE, 5NZ4, 5NZE, 5NZF, and 5NZN.

## 3. Results

### 3.1. The H275Y Mutation Impairs Neuraminidase Activity and Substantially Reduces Susceptibility to Oseltamivir

We used a standard fluorometric *in vitro* activity assay to investigate the impact of the I223V, S247N, and H275Y single mutations and the I223V/H275Y and S247N/H275Y double mutations reported in patients [27,42] on the catalytic activity of NA2009 [44]. The NA variants were cloned, recombinantly expressed in Drosophila Schneider S2 cells [45], purified, and kinetically characterized.

We analyzed the ability of NA variants to hydrolyze fluorometric substrate 4-MUNANA to determine the impact of amino acid substitutions on Michaelis–Menten (*K*_m_) constants and *k*_cat_ values (Table 2). Most of the mutated NAs retained *K*_m_ values almost identical to that of wild-type [62], while the S247N/H275Y double mutation resulted in a three-fold decrease in substrate-binding ability. However, we observed a much greater effect on the *k*_cat_ values, which were 1.5- to 20-fold lower for mutants compared to wt NA2009. The resulting catalytic efficiencies (*k*_cat_/*K*_m_) of the single mutants I223V and S247N were only moderately decreased. The catalytic efficiencies of the H275Y, I223V/H275Y, and S247N/ H275Y variants were 4–6% of the wt value, illustrating the significant effect of the H275Y mutation on the enzyme’s catalytic activity.

The sensitivities of the mutants to oseltamivir were determined by kinetic analysis and compared to that of wt NA2009. Analysis of the inhibition data (Table 2) revealed that while *K*_i_ values are increased only marginally for the I223V and S247N single mutants, H275Y substitution reduces susceptibility to oseltamivir by three orders of magnitude. *K*_i_ values for the I223V/H275Y and S247N/H275Y double mutants were 3900- and 9000-fold higher than the wt NA2009 value, respectively.

### 3.2. Neuraminidase Mutations Alter the Thermodynamic Parameters of Oseltamivir Binding

**Figure 1:**
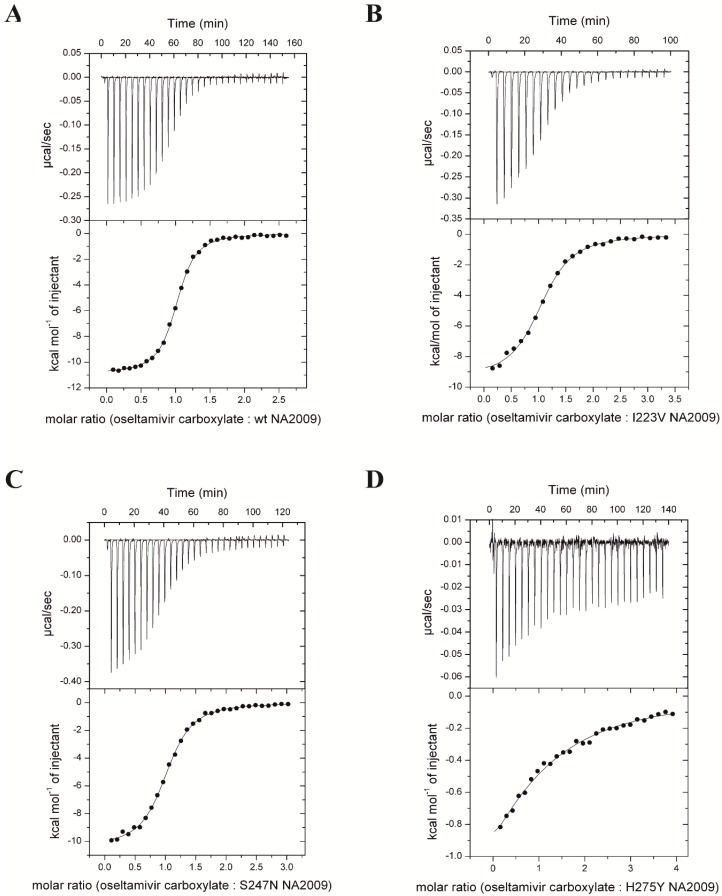
Thermodynamic analyses of oseltamivir binding. Isothermal titrations of oseltamivir carboxylate to the catalytic domain of wild-type NA2009 (**A**), I223V NA2009 (**B**), S247N NA2009 (**C**), and H275Y NA2009 (**D**) performed in 50 mM MES, pH 6.15, 150 mM NaCl, 10 mM CaCl_2_ at 25 °C.

We used isothermal titration calorimetry to monitor the binding of oseltamivir carboxylate to wt and mutated NA2009 (Table 3 and Figure 1). We could not perform titrations against double-mutants since the expression yield of recombinant protein was not sufficient for proper estimation of *K*_d_ values that are expected to be higher than for H275Y mutant. Titrations performed in buffers with different enthalpies of ionization (MES and ACES) yielded the same binding enthalpies, indicating that under the experimental conditions, there is no net proton transfer coupled to oseltamivir carboxylate binding to NA2009. Stoichiometries of the complexes were close to 1 (one inhibitor to one NA subunit). The dissociation constant (*K*_d_) of oseltamivir binding to wt NA2009 was 140 nM, which corresponds to a Gibbs energy of binding of −9.4 kcal mol^−1^. Oseltamivir bound to NA with a large favorable enthalpic contribution of −10.8 kcal mol^−1^ and slightly unfavorable entropic contribution of 1.5 kcal mol^−1^. The mutations in the resistant NAs decreased the binding affinity of oseltamivir by factors of 4 (I223V mutant), 3 (S247N mutant), and 95 (H275Y mutant).

Compared to wt, the I223V and S247N mutants had minor differences in the thermodynamic parameters of inhibitor binding. Oseltamivir carboxylate bound to these mutants with less favorable enthalpy changes of 1.4 kcal mol^−1^ and 0.4 kcal mol^−1^, respectively. The enthalpy change in I223V NA2009 was partly compensated by a more favorable entropic contribution of 0.6 kcal mol^−1^. The entropic contribution of oseltamivir binding to S247N NA2009 was almost unaffected; the value was within experimental error of the wt value.

For the H275Y single mutant, the enthalpy change for oseltamivir binding was substantially affected and was 8.7 kcal mol^−1^ less favorable than the wt value. This change was only partially compensated by a more favorable entropic contribution of 6 kcal mol^−1^ (referenced to the wt value).

### 3.3. Crystal Structures Help Elucidate the Mechanism of Oseltamivir Resistance

**Figure 2:**
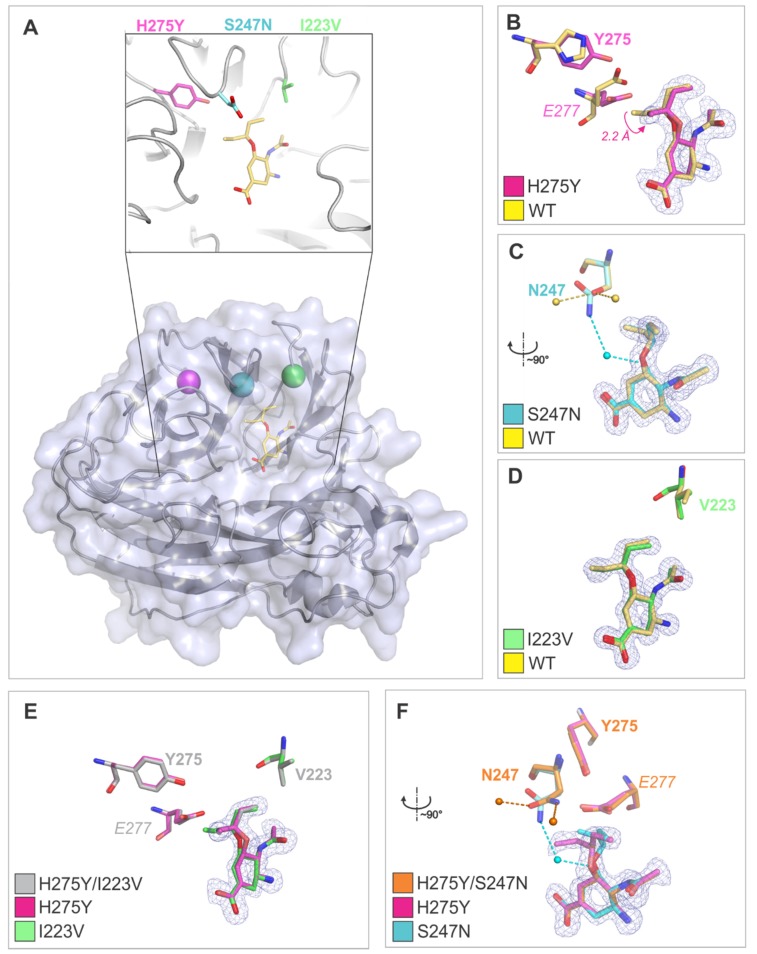
Crystal structures of mutant neuraminidase variants with oseltamivir. (**A**) Overall NA2009 structure with oseltamivir bound in the active site. Positions of the substituted amino acid residues are highlighted. Panels (**B**–**D**) show comparisons of the wild type enzyme active site with structures of enzymes carrying single amino acid substitutions. Panels (**E**,**F**) depict comparisons of the active sites of enzymes carrying single amino acid substitutions with variants containing two substitutions. The 2 *F*_O_-*F*_C_ electron density map for oseltamivir is contoured at 1 σ. Substituted residues and residues with large structural changes are shown as sticks and labeled. Carbon atoms and water molecules of wt NA2009 are colored golden, H275Y magenta, I223V green, S247N turquoise, H275Y/I223V grey, and H275Y/S247N orange. Water molecules are represented by spheres with hydrogen bonds shown as dashed lines.

To decipher the structural mechanism of resistance, we determined crystal structures of NA2009 mutants in complex with oseltamivir carboxylate. Three variants (H275Y, H275Y/I223V, and H275Y/S247N NA2009) crystallized in the monoclinic space group with four molecules in the asymmetric unit, representing the tetrameric biological unit. Two variants (I223V and S247N single mutants) crystallized in the centered orthorhombic space group with two molecules in the asymmetric unit; the tetrameric biological unit could be reconstructed from two symmetrically related dimers. The RMSD value for superposition of the Cα atoms in the protein chains present in the asymmetric unit did not exceed 0.13 Å, which is lower than the value observed for identical structures [63]. Thus, A chains were used in structural analyses. The non-protein electron density in the active site of all enzyme variants was of good quality and allowed modeling of oseltamivir carboxylate with full occupancy in all protein chains (Figure 2).

Crystal structures of enzymes containing the H275Y substitution either alone or in combination with other mutations show that the bulkier tyrosine side chain pushes the side chain of the neighboring E277 into the binding site, affecting the binding pose of oseltamivir. Specifically, the position of the pentyloxy substituent of oseltamivir changes relative to that in the wt enzyme; the shift in position of the terminal C atom (C91) is more than 2 Å (Figure 2B). The crystal structure provides a direct explanation for the more than 1000-fold reduction in binding affinity for oseltamivir in variants containing the H275Y substitution. A similar effect on oseltamivir binding was previously observed for the H275Y substitution in H5N1 avian influenza neuraminidase [30].

On the other hand, the effects of the I223V and S247N substitutions are less dramatic on the structural level. No change in oseltamivir pose was observed; the RMSD for superposition of oseltamivir atoms in the wt NA2009 structure (PDB code 3TI6), I223V variant, and S247N variant was 0.74 Å.

The I223V substitution did not induce any structural changes in the neighboring residues; however, the substitution for a smaller side chain results in enlargement of the volume of the enzyme active site pocket and loss of Van der Waals contacts with the pentyloxy substituent of oseltamivir. The methyl group of the I223 side chain in the wt enzyme forms a dispersion bond (C-C distance of 4.1 Å) with the methyl group of oseltamivir. This interaction is not present in the I223V mutant, where the closest C-C distance between V223 and oseltamivir methyl groups is 5.0 Å. The energy of this interaction can be estimated as ~1 kcal/mol [64]. This explains the 30-fold increase in *K*_i_ value for oseltamivir upon I to V substitution.

Residue 247 is located at the edge of the enzyme active site. As its side chain is exposed to the solvent, substitution of serine with the larger asparagine (S247N mutation) does not induce changes in the protein structure; the RMSD for superposition of 390 Cα with the wt structure was 0.133 Å. Nevertheless, changes in the structure of the hydration shell were observed. Namely, N247 is involved in a water-mediated contact with the oxygen atom in the pentyloxy substituent of oseltamivir, an interaction that is not present in the wt enzyme. S247 forms hydrogen bonds with two water molecules present only in the wt enzyme and not in the S247N mutant.

In enzyme variants containing H275Y in combination with the I223V or S247N mutation, an oseltamivir pose similar to that found in H275Y was observed (Figure 2B,E,F). The RMSD for superposition of oseltamivir atoms in the H275Y, H275Y/I223V, and H275Y/S247N NA2009 variant was below 0.11 Å. We conclude that structural changes caused by the individual mutations have a coacting effect on increase of oseltamivir resistance, reflected in the more than three-fold increase in *K*_i_ in H275Y/I223V NA2009 compared to H275Y NA2009.

In the H275Y/S247N variant, the water-mediated contact with the oxygen atom between the N247 side-chain and pentyloxy substituent of oseltamivir seen in S247N NA2009 does not form due to the change in position of the pentyloxy substituent caused by the H275Y substitution. The dramatic effect of the H275Y/S247N double mutation on susceptibility to oseltamivir (8-fold and 500-fold increase in *K*_i_ compared to the H275Y and S247N single mutants, respectively) can thus be attributed to the combined influence of the change in oseltamivir pose and alterations in the hydration shell structure.

## 4. Discussion

The neuraminidase inhibitor oseltamivir is an effective anti-influenza drug and is used extensively worldwide. However, oseltamivir resistance has become a critical problem due to the rapid emergence of oseltamivir-resistant strains of the N1-subtype. In this study, we applied a set of experimental techniques using recombinant NA variants to understand the molecular basis of development of oseltamivir resistance in the viral N1 neuraminidase NA2009. To date, mutations associated with a significant decrease in susceptibility to oseltamivir have been reported at only a few positions adjacent to the NA active site [30,65,66,67]. We investigated the impact of mutations at NA2009 residues 223, 247, and 275 and achieved good agreement between kinetic data, thermodynamic parameters of oseltamivir binding obtained by ITC, and data from X-ray crystallographic structures.

Our kinetic studies showed significantly lower enzymatic *k*_cat_ values (1.5 to 20-fold) for mutants compared to wt NA2009, while all variants preserved the ability to bind substrate. We observed only moderately decreased catalytic efficiency (*k*_cat_/*K*_m_) for the single mutants I223V and S247N, and much lower values for all variants containing the H275Y substitution (only 4–6% of the wt value for the H275Y, I223V/H275Y, and S247N/H275Y mutants). Compared to wt, the sensitivity of the H275Y single mutant to oseltamivir decreased considerably (more than 1000-fold increase in *K*_i_). The effects of the I223V and S247N single mutations were more modest (approximately 30- and 20-fold increases in *K*_i_, respectively). The double mutations I223V/H275Y and S247N/H275Y led to dramatic increases in *K*_i_ values (3900- and 9000-fold higher than wt, respectively).

These data are consistent with thermodynamic ITC data showing minor changes in the parameters of oseltamivir binding to the I223V and S247N single mutants compared to wt. The substantial decrease in strength of oseltamivir binding to the H275Y mutant is caused by a significant loss of binding enthalpy, which is partially compensated by a gain in binding entropy.

To structurally explain the effect of the amino acid exchanges on oseltamivir resistance, we performed X-ray structure analysis of five oseltamivir-resistant NA variants in complex with the inhibitor. To our knowledge, structural analyses of these mutant variants are the only reported on the background of the pandemic NA2009. In all crystal structures containing a H275Y mutation, the binding pose of oseltamivir was altered due to the bulkier, polar tyrosine side chain pushing E277 into the active site. This observation explains the 1100-fold reduction in the binding affinity of the H275Y mutant to oseltamivir and is in agreement with previous structural analysis of the H275Y variant of avian influenza neuraminidase H5N1 by Collins and co-workers [30]. However, we did not confirm the loss of a hydrogen bond between R152 and the oseltamivir acetyl group, which was proposed by Le and co-workers based on molecular dynamics simulations as an additive molecular mechanism conferring drug resistance in H5N1 and H1N1 strains bearing H275Y mutations [68].

The I223V and S247N substitutions did not have a dramatic effect on the oseltamivir binding pose. Rather, small structural alterations—such as loss of a dispersion bond with oseltamivir in the V223 mutant and changes in the hydration shell in the N247 mutant—appear responsible for increases in *K*_i_.

Taken together, our kinetic, thermodynamic, and structural results show that while the I223V and S247N substitutions alone confer only mild oseltamivir resistance, combination with the major neuraminidase mutation H275Y leads to dramatic impairment of inhibition potency that can be attributed to the additive structural and hydration shell changes.

We hope that a detailed understanding of the origins of resistance at the molecular level will aid development of more potent compounds that are effective against oseltamivir-resistant NAs.

## Figures and Tables

**Table 1:** Crystal data and diffraction data collection and refinement statistics.

NA2009 Variant	H275Y	I223V	S247N	H275Y/I223V	H275Y/S247N
PDB Code	5NWE	5NZ4	5NZE	5NZF	5NZN
**Data collection statistics**					
Space group	*P*2_1_	*C*222_1_	*C*222_1_	*P*2_1_	*P*2_1_
Cell parameters (Å; °)	a = 85.47 b = 126.60 c = 96.56 α = γ = 90 β = 93.49	a = 119.46 b = 137.58 c = 118.61 α = β = γ = 90	a = 118.67 b = 136.59 c = 119.14 α = β = γ = 90	a = 85.65 b = 127.22 c = 96.79 α = γ = 90 β = 92.93	a = 85.78 b = 127.54 c = 96.77 α = γ = 90 β = 93.24
Number of molecules in AU	4	2	2	4	4
Wavelength (Å)	0.918	0.918	1.54	0.918	0.918
Resolution (Å)	48.2–2.00 (2.12–2.00)	44.92–1.37 (1.41–1.37)	38.00–1.69 (1.75–1.69)	45.18–1.75 (1.81–1.75)	48.31–1.73 (1.79–1.73)
Number of unique reflections	137,306 (21,721)	198,814 (19,191)	101,433 (8959)	207,181 (20,679)	214,541 (20,953)
Redundancy	3.0 (3.0)	3.4 (3.4)	4.8 (3.6)	3.4 (3.2)	3.4 (3.2)
Completeness (%)	98.8 (96.9)	96.6 (93.9)	93.9 (83.6)	99.6 (99.8)	99.4 (97.3)
R_merge_^a^	13.9 (62.1)	9.6 (81.5)	9.3 (68.2)	11.4 (73.3)	11.0 (73.1)
Average I/σ(I)	7.6 (2.0)	10.5 (1.6)	11.6 (2.0)	9.0 (1.8)	9.4 (1.8)
Wilson B (Å^2^)	27.11	11.9	16.6	16.6	15.7
**Refinement statistics**					
Resolution range (Å)	48.19–2.00 (2.05–2.00)	44.92–1.37 (1.41–1.37)	38.00–1.69 (1.75–1.69)	45.18–1.75 (1.81–1.75)	48.31–1.73 (1.79–1.73)
No. of reflections in working set	135,202 (9455)	198,810 (19,192)	101,425 (8959)	207,153 (20,678)	214,496 (20,952)
No. of reflections in test set	2100 (147)	1989 (192)	2028 (179)	2176 (217)	2253 (220)
R value (%) ^b^	23.6 (28.4)	14.5 (30.7)	19.1 (25.8)	22.0 (29.2)	23.4 (29.6)
R_free_ value (%) ^c^	27.7 (34.3)	17.0 (33.8)	21.6 (27.0)	26.3 (31.9)	26.7 (32.5)
RMSD bond length (Å)	0.014	0.017	0.019	0.017	0.019
RMSD angle (°)	1.60	1.77	1.79	1.77	1.86
Number of atoms in AU	13,704	7384	7033	13,691	13,692
Number of protein atoms in AU	12,008	6067	6031	12,005	12,025
Number of water molecules in AU	1380	1084	795	1337	1382
Mean B value all/protein/waters (Å^2^)	23.9/22.7/31.0	14.3/11.2/28.6	17.7/15.8/28.6	19.4/18.1/27.4	18.2/17.0/26.0
**Ramachandran plot statistics** **^d^**					
Residues in favored regions (%)	95.5	96.2	96.8	96.2	96.0
Residues in allowed regions (%)	4.4	3.8	3.2	3.6	4.0

The data in parentheses refer to the highest-resolution shell. ^a^ R*merge* = ∑*hkl*((n/(n − 1))1/2∑j|*I*i(*hkl*)—〈*I*(*hkl*)〉|)/∑*hkl*∑i *I*i(*hkl*), where the *I*i(*hkl*) is an individual intensity of the ith observation of reflection hkl and 〈I(hkl)〉 is the average intensity of reflection hkl with summation over all data. ^b^ R value = ||*F*o| − |*F*c||/|*F*o|, where *F*o and *F*c are the observed and calculated structure factors, respectively. ^c^ R_free_ value is equivalent to R value but is calculated for the reflections chosen at random and omitted from the refinement process [53]. ^d^ as determined by Phenix [54].

**Table 2:** Kinetic parameters of recombinant wild-type neuraminidase NA2009 and oseltamivir-resistant mutants calculated as means and s.d. from two independent experiments

NA Mutation ^a^	*K*_m_ [mM]	*k*_cat_ [s^−1^]	*k*_cat_/*K*_m_ [M^−1^ s^−1^]	*K*_i_ [nM]	Fold *K*_i_^b^
-	1.1 ± 0.03	0.90 ± 0.01	820 ± 30	24 ± 4	1
H275Y	1.1 ± 0.2	0.054 ± 0.004	48 ± 11	27,000 ± 2000	1100
I223V	1.2 ± 0.2	0.60 ± 0.05	510 ± 110	700 ± 100	29
S247N	1.1 ± 0.1	0.17 ± 0.01	160 ± 20	450 ± 40	19
H275Y/I223V	1.3 ± 0.3	0.045 ± 0.005	35 ± 10	94,000 ± 7000	3900
H275Y/S247N	3.3 ± 0.5	0.10 ± 0.01	31 ± 6	220,000 ± 4000	9000

^a^ All mutations are presented in N1 numbering. ^b^ Compared with that of wt NA2009 [56].

**Table 3:** Thermodynamic parameters of oseltamivir carboxylate binding to wild-type NA2009 and drug-resistant mutants (I223V, S247N, H275Y). Binding values are means and s.d. of two independent experiments performed in 50 mM MES (or ACES), pH 6.15, 150 mM NaCl, 10 mM CaCl_2_ at 25 °C.

	Stoichiometry	ΔG	ΔH	−T·ΔS	*K*_d_	Fold
NA Mutation ^a^	Inhib./NA Unit	kcal mol^−1^	kcal mol^−1^	kcal mol^−1^	nM	*K*_d_^b^
**oseltamivir carboxylate**						
-	1.05 ± 0.05	−9.4 ± 0.1	−10.8 ± 0.1	1.5 ± 0.2	140 ± 20	1
I223V	1.06 ± 0.01	−8.6 ± 0.1	−9.4 ± 0.1	0.9 ± 0.2	520 ± 30	4
S247N	1.02 ± 0.01	−8.7 ± 0.03	−10.4 ± 0.1	1.7 ± 0.1	410 ± 20	3
H275Y	0.96 ± 0.03	−6.7 ± 0.03	−2.1 ± 0.04	−4.6 ± 0.1	13,200 ± 600	95

^a^ All mutations are presented in N1 numbering. ^b^ Compared with that of wt NA2009 [56].
